# Supplementary figures and images for: Estimating ectopic beat probability with simplified statistical models that account for experimental uncertainty
Source: PLoS Comput Biol. 2021 Oct 19;17(10):e1009536. doi: 10.1371/journal.pcbi.1009536 (PMC8577785; doi:10.1371/journal.pcbi.1009536)

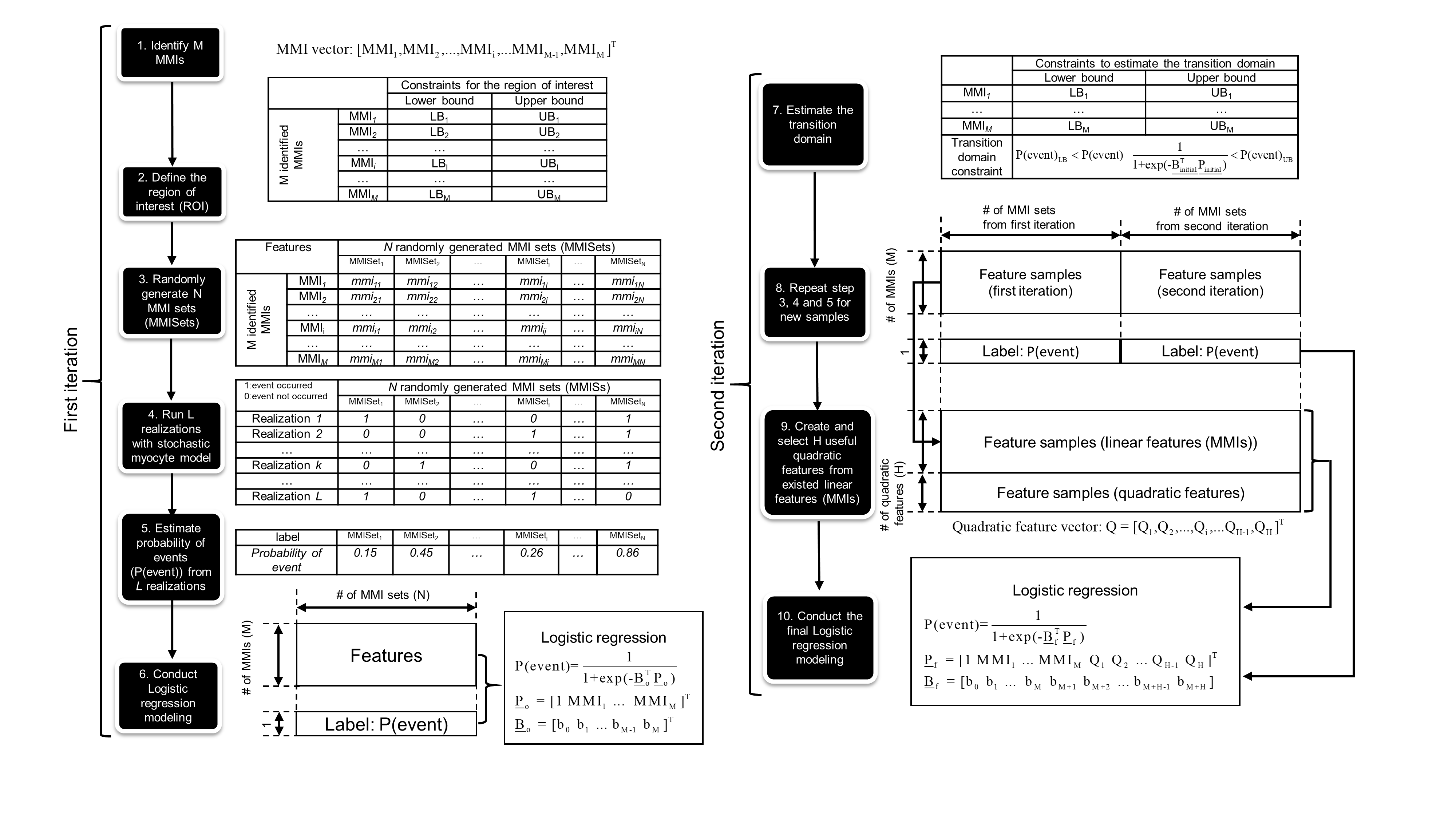

Supplement: S1 Fig — (TIF) [file pcbi.1009536.s001.tif]

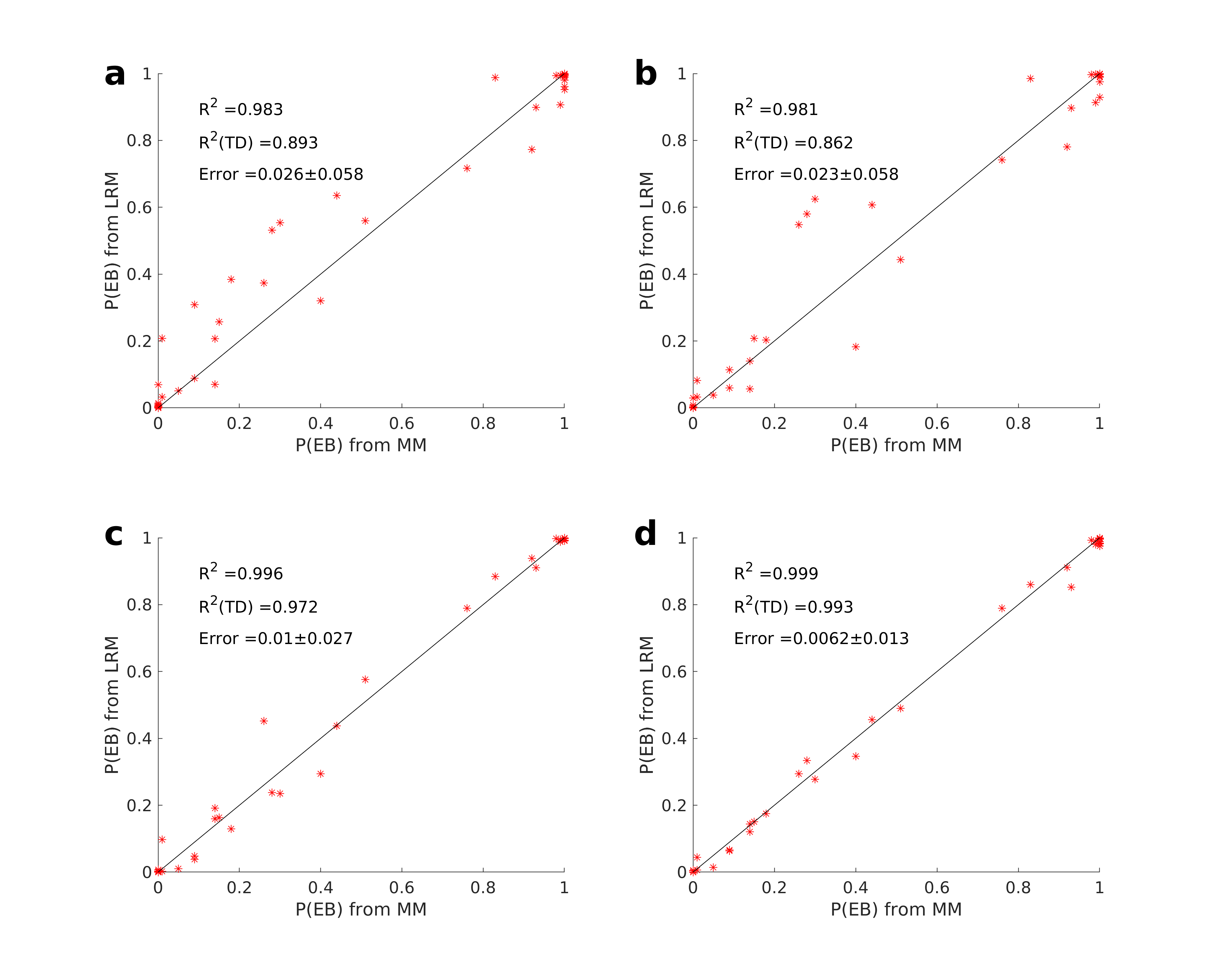

Supplement: S2 Fig — Performance of each LRM is evaluated on the same test set which consists of 100 myocyte model input (MMI) sets for all models. Each LRM has a unique training strategy. A) LRM trained with only linear features (excluding quadratic features) and only 100 MMI sets from only the first iteration. B) LRM trained with only linear features (excluding quadratic features) and 200 MMI sets (100 sets in the first iteration + 100 transition domain sets in the second iteration). C) LRM trained with linear features and quadratic features on 100 MMI sets from only the first iteration. D) Reproduction of Fig 1E, LRM trained with linear features and quadratic features and 200 MMI sets (100 sets in the first iteration + 100 transition domain sets in the second iteration). Linear features and quadratic features used in A-D are given in Table 1. First iteration training data was identical for all LRMs and second iteration training data was identical for LRMs in panels B and D. R2 for the entire region of interest and transition domain as well as the average error are reported. (TIF) [file pcbi.1009536.s002.tif]

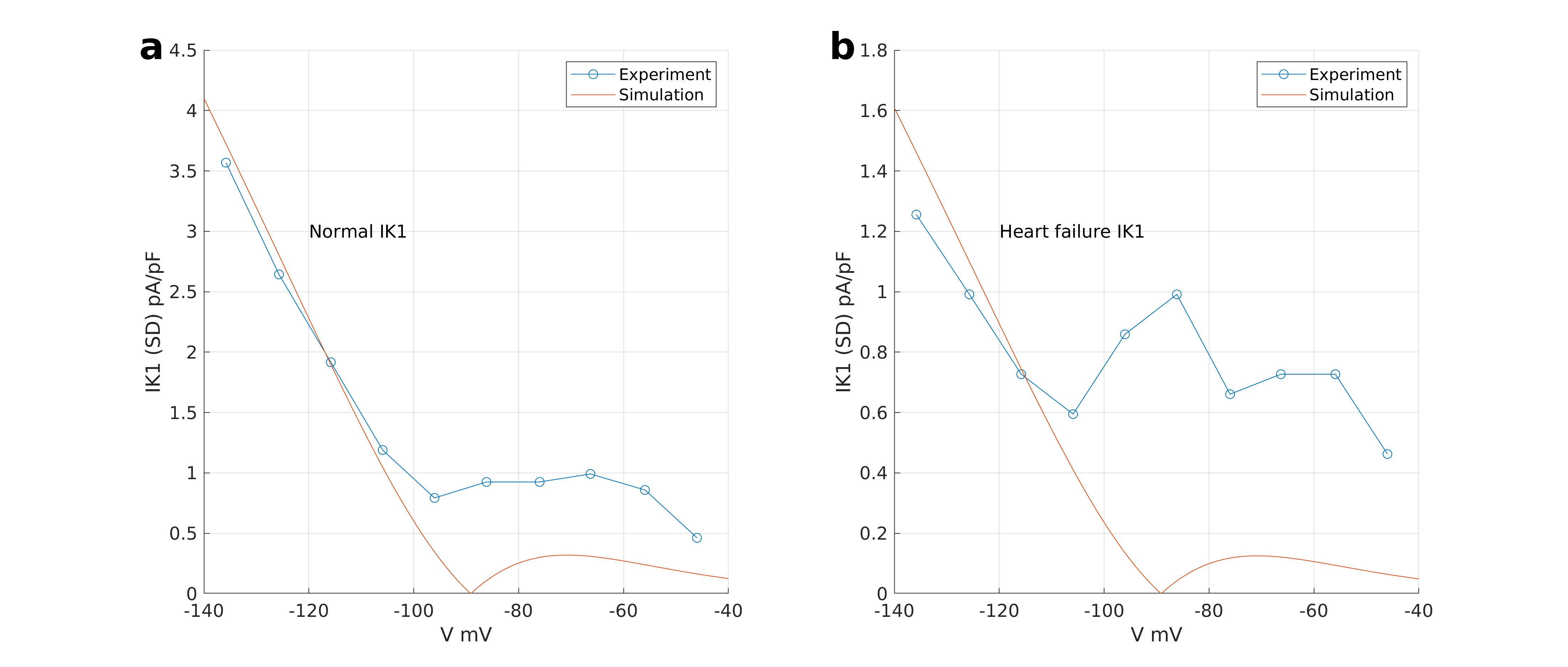

Supplement: S3 Fig — Experimental IK1 I-V relationship for normal and Heart failure (HF) is measured and showed in Fig 5C of Pogwizd et. al. In the experimental protocol, IK1 is measured in response to 500-ms steps from a holding potential of -30 mV to test potentials in the range of -120 mV to +40 mV in 6 normal cells and 6 HF cells. The detail of the protocol is described in Pogwizd et. al. Error bars represent standard error (SE). (A) Best fit of the standard deviation (SD) for the normal IK1 using the same voltage clamp protocol. (B) Best fit of the SD for the HF IK1 using the same voltage clamp protocol. (TIF) [file pcbi.1009536.s003.tif]
