# Supplementary material for: Estimating ectopic beat probability with simplified statistical models that account for experimental uncertainty
Source: PLoS Comput Biol. 2021 Oct 19;17(10):e1009536. doi: 10.1371/journal.pcbi.1009536 (PMC8577785; doi:10.1371/journal.pcbi.1009536)
Supplement: S1 Table — (DOCX) [file pcbi.1009536.s004.docx]

**S1 Table. Sub-model performance on the 100 MMI test set**

| Included features | R^2^ | error |
| --- | --- | --- |
| Top 1 feature | 0.511 | 0.267±0.203 |
| Top 2 features | 0.786 | 0.133±0.175 |
| Top 3 features | 0.779 | 0.133±0.180 |
| Top 4 features | 0.773 | 0.130±0.185 |
| Top 5 features | 0.768 | 0.131±0.188 |
| Top 6 features | 0.758 | 0.119±0.202 |
| Top 7 features | 0.995 | 0.013±0.032 |
| Top 8 features | 0.999 | 0.007±0.016 |
| Top 9 features | 0.998 | 0.008±0.018 |
| All 10 features | 0.999 | 0.006±0.013 |
