# Supplementary material for: Estimating ectopic beat probability with simplified statistical models that account for experimental uncertainty
Source: PLoS Comput Biol. 2021 Oct 19;17(10):e1009536. doi: 10.1371/journal.pcbi.1009536 (PMC8577785; doi:10.1371/journal.pcbi.1009536)
Supplement: S1 Text — (DOCX) [file pcbi.1009536.s005.docx]

# **Supporting Information**

## **S1 Text. Supporting description of logistic regression model and entropy.**

**Logistic regression model**. S1 Fig shows the workflow for building a two-iteration logistic regression model. In the first iteration, the first step is to identify M MMIs: MMI_i_, i = 1, 2, …, M-1, M. Four MMIs are chosen for the EB study as described in the Method. The region of interest (ROI) for each MMI, representing the biophysical meaningful range of the values of MMI_i_, is then determined based on experiments and defined by an upper bound (UB_i_) and a lower bound (LB_i_). The ROI of the full set of MMIs makes up a M-dimensional MMI space Ω ∈ R^M^. We randomly generate N MMI sets (MMISets) MMISet_j_ ∈ Ω, j = 1, 2, …, N with a uniform distribution. The j^th^ MMI set MMISet_j_ contains M MMI values, where MMISet_j_ = [mmi_1j_, mmi_2j_, …, mmi_Mj_]^T^. Stochastic myocyte model (MM) simulations are performed L times for each MMISet_j_ to obtain L realizations per MMI set. The detailed MM simulation protocol is described in Methods. The output of the MM for each MMISet_j_ will be a vector of length L with binary elements where each element indicates whether (= 1) or not (= 0) the event of interest (in this case EB) occurred. A probability of the event occurrence, P_j_(event), is estimated from the output vector for each MMISet_j_. Then, we performed logistic regression on all MMI sets and their corresponding event probabilities. More specifically, the MxN feature matrix is [MMISet_1_, MMISet_2_, …, MMISet_N_] and the 1xN label vector is [P_1_(event), P_2_(event), …, P_N_(event)]. The logistic equation takes the form

$\text{P(event)=}\frac{1}{\text{1+exp(-}\underline{\text{B}}_{\text{o}}^{\text{T}}\underline{\text{P}}_{\text{o}}\text{)}}$ (S1)

, where P_o_ = [MMI_1_, MMI_2_,…, MMI_M_] is the feature vector input for the logistic equation, B_o_ = [b_0_, b_1_,…, b_M_] is the vector of weights and P(event) is the predicted event probability from the logistic equation. In summary, in this first iteration (step 1 – 6 in S1 Fig), we perform a logistic regression on the feature matrix and the label vector, and this yields our first estimation of logistic equation weights (B_o_).

After the first iteration, we use B_o_ with S1 Eq to create an additional constraint in addition to that imposed by the ROI in step 2 of S1 Fig to estimate the transition domain (TD)

$\text{P(event)}_{\text{LB}}<\text{P(event)=}\frac{1}{\text{1+exp(-}\underline{\text{B}}_{\text{o}}^{\text{T}}\underline{\text{P}}_{\text{o}}\text{)}}<\text{P(event)}_{\text{UB}}$ (S2)

where P(event)_LB_ is the lower bound for P(event) and P(event)_UB_ is the upper bound for P(event). In this study, we chose P(event)_LB_ = 0.01 and P(event)_UB_ = 0.99. We repeat steps 3, 4, and 5 described in S1 Fig on the estimated TD from S2 Eq. Therefore, we obtain an additional N newly generated MMISets. Combining with the MMISets generated in the first iteration, the new feature matrix will then be [MMISet_1_, …, MMISet_N_, MMISet_N+1_, …, MMISet_2N_]. Label vectors becomes [P_1_(event), …, P_N_(event), P_N+1_(event), …, P_2N_(event)]. In order to further improve the model performance, we derived quadratic features from the MMIs in the form of MMI_i1_*MMI_i2_, i1, i2 = 1, 2, …, N. Besides linear features (MMIs), we enumerated all possible combinations of quadratic features (Qs) and choose those with the minimum of the consistent Akaike information criterion (CAIC) [1] for our model. See selection strategy for quadratic features in Methods. After adding H selected quadratic terms into feature matrix, we conduct logistic regression again with the updated feature matrix and label vector to obtain the second iteration logistic equation

$\text{P(event)=}\frac{1}{\text{1+exp(-}\underline{\text{B}}_{\text{f}}^{\text{T}}\underline{\text{P}}_{\text{f}}\text{)}}$ (S3)

where P_f_ = [MMI_1_, MMI_2_, …, MMI_M_, Q_1_, Q_2_, …, Q_H_] is the feature vector (Q_1_, …, Q_H_ are quadratic features) and B_f_ = [b_0_, b_1_,…, b_M+H_] is the vector of weights. S3 Eq is our final simplified model which is referred to simply as the logistic regression model (LRM).

**Simulation protocol**. To speed up the simulation, we stopped the simulation at an early phase if the membrane potential profile satisfied either of three conditions. 1) If the membrane potential exceeds 0mV, we stop the simulation and assume the ectopic beat has occurred. 2) If the membrane potential at 600ms < -85mV, we stop the simulation and assume that the ectopic beat does not occur within 800ms. 3) If the membrane potential falls below its recent local maximum value by more than 5mV, we stop the simulation and assume that no ectopic beat occurred.

**Entropy.** Entropy, a measure of the uncertainty of random variables (RVs) [2], is more appropriate than the variance to assess uncertainty for multimodal distributions [3]. The RV with larger uncertainty will have a larger entropy. Given the fact that the P(EB) distribution can become bimodal, we use the entropy to quantify P(EB) uncertainty. We separated the P(EB) distributions into 100 bins where the interval for *i*th bin is [0.01*i, 0.01*i+0.01], i=0, 1, …, 99. Assume that the *i*th bin’s sample frequency is freq_i_, the entropy of P(EB) distribution is

$Entropy=-\sum_{i=0}^{99} freq_{i}\ln(freq_{i})$ (S4)

**Uncertainty analysis.** If no quadratic features are considered in the LRM. The distribution of the probability of ectopic beat (P(EB)) can be calculated analytically, and is the logit-normal distribution [4]. If we assume each MMI is an independent random variable, we have the probability density function of P(EB)

$f(P(EB);\mu,\sigma)=\frac{1}{\sigma\sqrt{2\pi}}\frac{1}{P(EB)(1-P(EB))}e^{-\frac{\left(\ln\left(\frac{P(EB)}{1-P(EB)}\right)-\mu\right)^{2}}{2\mu^{2}}}$ (S5)

, where $\mu=b_{0}+b_{1}\mu_{1}+b_{2}\mu_{2}+...+b_{M}\mu_{M}$, $\mu_{i}$ is the mean of *i*th MMI, $\sigma=\sqrt{b_{1}^{2}\sigma_{1}^{2}+b_{2}^{2}\sigma_{2}^{2}+...+b_{M}^{2}\sigma_{M}^{2}}$, $\sigma_{i}$ is the standard deviation of *i*th MMI.

# **References**

1. Bozdogan H. Model selection and Akaike's information criterion (AIC): The general theory and its analytical extensions. Psychometrika. 1987;52(3):345-70.

2. Wang QA. Probability distribution and entropy as a measure of uncertainty. Journal of Physics A: Mathematical and Theoretical. 2008;41(6):065004.

3. Smaldino PE. Measures of individual uncertainty for ecological models: Variance and entropy. Ecological modelling. 2013;254:50-3.

4. Atchison J, Shen SM. Logistic-normal distributions: Some properties and uses. Biometrika. 1980;67(2):261-72.
