# Supplementary material for: Estimating ectopic beat probability with simplified statistical models that account for experimental uncertainty
Source: PLoS Comput Biol. 2021 Oct 19;17(10):e1009536. doi: 10.1371/journal.pcbi.1009536 (PMC8577785; doi:10.1371/journal.pcbi.1009536)
Supplement: Attachment — Submitted filename: response letter.docx [file pcbi.1009536.s006.docx]

Thank you for the opportunity to submit a revised draft of the manuscript “Estimating Ectopic beat Probability with Simplified Statistical Models that Account for Experimental Uncertainty”. We appreciate the time and effort that you and reviewers dedicated to providing feedback on our manuscript and are grateful for the insightful comments. We highlighted all changes in the revised manuscript. In this letter, a point-by-point response to reviewer’s comments and concerns are made. For easier reading, we use black for original reviewer comments, blue for our responses, and green for changes that we have made to the manuscript. Line numbers indicated here correspond to the line numbers appearing in the revised manuscript (tracked version) under the All Markup mode.

**Reviewer’s Comments to the Authors:**

**Reviewer #1**

The study by Jin et al is an interesting study that presents the utility of a statistical model (specifically a logistic regression model) to predict the probability of an ectopic beat in an isolated cardiac cell, based on a detailed biophysical model that incorporates stochastic calcium cycling. The study is of interest to the computational biology community and the manuscript is well-written. However, I have some concerns for the authors to address:

**Major:**

1. The rationale of the specific 10 features used in the LRM is unclear as presented in the main text, specifically why only certain quadratic terms are used, and if some feature selection process was chosen. Eventually I found this detail in the Supporting Methods – that the LRM was chosen based on CAIC – but the details of this process need to be included in the main text.

Thank you for pointing this out. The reviewer is correct.

As suggested by the reviewer, we added a detailed description of the quadratic feature selection process in the Methods (Line 499 – 507). Additionally, we also give a brief description of quadratic feature selection where we first mentioned it in the Results (Line 143 – 145).

Regardless, why did the authors choose these specific 4 MMIs to consider for the LRM? Presumably, the model is also highly dependent on other inputs, specifically Ca cycling related parameters. This seems to bias the analysis that could be missing other parameters equally or more important in EB triggers.

We agree with your comment that other model parameters may have significant impact on the EB triggers. However, the discovery of all relevant parameters that influence P(EB) is not a central goal of this study, rather we aim to communicate the method by which we perform arrhythmia uncertainty analysis for a chosen set of parameters and model inputs. All model parameters included in this study have been previously well-studied. Walker et al. (PMID: 29145393) have quantitatively studied the relationship between probability of ectopic beat P(EB) and initial state of cytosolic Ca^2+^ ([Ca]_i_^ini^), initial state of Ca^2+^ in sarcoplasmic reticulum ([Ca]_SR_^ini^) and conductance of inward rectifier K^+^ current (G_K1_). Paavola et. al. (PMID: 17347175) also demonstrated the positive correlation between RyR opening rate with DAD induction and Ca^2+^ waves. The novel contribution of this paper is to show that the LRM-based approach makes it possible to efficiently study and analyze the uncertainty associated with a chosen set of model parameters.

We agree that there is a possibility that some MMIs not considered in this particular study will be equally or more important in determining the distribution of P(EB). Regarding the feature ranking result, when we say that “Feature [Ca^2+^]_SR_ is the most important” in Line 140, it specifically means that Feature [Ca^2+^]_SR_ is the most important among the four myocyte model inputs (MMIs) and their derived quadratic features investigated in this study.

Thus, we added a subsection in the discussion to clarify the applicable scope of the ranking in Line 357-361.

2. It seems like the analysis/simulations presented will also produce predictions for the probability of DADs, in addition to EBs. Can the authors compare the ability to use the LRM to predict P(DADs)? It would be valuable to compare conditions that lead to DADs with conditions that lead to EBs. Additionally, the LRM framework would enable particularly useful predictions. For example, how does uncertainty in DADs relate to uncertainty in EBs? Similarly, does uncertainty in DADs relate to changes in P(EB)? Does DADs uncertainty decrease as P(EB) increases?

We agree that it would be interesting to use this approach for predicting delayed after-depolarizations (DADs). However, defining the probability of a DAD (P(DAD)) is a difficult task given the fact that, unlike the ectopic beat, a DAD is not an all-or-none phenomenon. The amplitude of DADs varies over a wide range. To show this, we calculated the amplitude of DADs for all 300 MMI sets (200 training MMI sets and 100 test MMI sets). Specifically, for every parameter set within the 300 MMI sets, we calculated the mean value of the maximum voltage achieved across all realization in which a DAD with no ectopic beat occurred. We then calculated the difference between the mean value of the maximum voltage and the resting potential (-93mV), which gives the DAD amplitude. Across the 300 MMI sets, the DAD amplitude varies from ~0 mV – 30 mV. Thus, to calculate a probability measure of DADs, we need to set a voltage threshold (in the range of 0 to 30 mV) and label the outcome as a DAD if the threshold is exceeded. Because there are infinitely many choices for such a voltage threshold, there remains ambiguity in the definition of DAD occurrence. Therefore, rather than defining P(DAD), we believe that it would be more interesting to predict the DAD amplitude. Walker et al. (PMID: 29145393) has developed an approach to predict the maximum voltage of stochastically occurring DADs, equivalent to the DAD amplitude.

Additionally, to some extent, the P(EB) prediction described in this paper also represents the probability of DADs exceeding a critical voltage threshold, where passing this critical threshold leads to the ectopic beat. We believe that this critical voltage threshold is a functionally relevant choice rather than arbitrarily choosing a voltage threshold for DADs.

We very much appreciate the suggestion to incorporate DAD prediction with the P(EB) prediction. It would certainly be very interesting to study the relationship between DAD and P(EB) as described in the last three questions raised in your comments, especially when we change the focus from P(DAD) to DAD amplitude. However, given the rich content of such a study, we believe that it can be an independent work and is beyond the scope of this study. Thus, we have discussed these ideas in the discussion as a part of our future work.

We added a subsection in Line 413-423 in the discussion to describe that predicting the DAD amplitude and analyzing the relationship between DAD amplitude and P(EB) as potential future work.

3. It is interesting that the top 5 terms in the LRM by weight only contain terms for Ca_SR and kRyR_sf. Did the authors consider the sub-model that only contains these terms and compare with the full 10 term model? The relative weights suggests that this model may be quite good at prediction as well.

To address reviewer’s comment and further extend the concept of the sub-model, we trained 9 sub-models which included the top *n* (for *n* = 1…9) features showed in Table 1 on the same 200 training MMI sets, respectively. We, then, obtained the R^2^ and error (mean ± SD) for all 9 sub-models on the same 100 test MMI sets (shown in S2 Table). The sub-model with top 5 features yields a R^2^=0.768 and the mean error of P(EB) = 0.131. In comparison, the mean error of P(EB) for the full model is 0.006. Thus, the sub-model with only [Ca^2+^]_SR_^ini^ and [Ca^2+^]_i_^ini^ cannot make satisfactory predictions. S2 Table shows that the sub-model with the top 7 features has mean error of P(EB) = 0.013 and R^2^=0.995, whereas the sub-model with the top 6 features has mean error P(EB) = 0.119 and R^2^ =0.758. This implies that the top 7 features are necessary to reproduce similar performance as that of the full model. Given the fact that model with the top 7 features is the smallest sub-model containing all four MMIs, it implies that all four MMIs are necessary to make a good prediction.

We added a subsection in the discussion Line 362 – 372 to discuss the sub-model results indicating that all four MMIs are required to make a good P(EB) prediction. We also added a table (S3 Table) to summarize the results from all sub-models.

4. The author’s note that their work builds of prior work from the Sobie lab, and it seems that prediction of an output distribution and uncertainty is one novel aspect of the authors’ extension. This could probably be highlighted to a greater extent.

Additionally, measurement of uncertainty could more broadly be applied to consider practical aspects of experimental measurements, by investigating parameter uncertainty in all inputs at once. For example, if there is parameter uncertainty in all inputs with a magnitude of 10%, how much uncertainty propagates to the P(EB) prediction? How much uncertainty is “tolerable” in measurements in order to make reasonable predictions of P(EB)?

We very much agree with your suggestions on the uncertainty analysis. We added a subsection in the Discussion Line 401 – 412 to emphasize the significance of our uncertainty analysis approach. We used your two questions as examples to show the potential applications.

Minor:

1. There are some terminology ambiguities regarding describing Ca_i and Ca_SR as parameters early in the paper. This is eventually clarified when the authors introduce the nomenclature of MMIs, which includes the initial conditions for these state variables, but this is unclear in the abstract and introduction. I would suggest defining a different notation for the initial conditions, e.g., Ca_i^0 and Ca_SR^0, to distinguish these values from the state variables.

We agree that it is better to make a different notation to describe the initial states of [Ca^2+^]_i_ and [Ca^2+^]_SR_. However, we found that adding superscript “0” will be confused with the quadratic terms. For example, ([Ca^2+^]_SR_^0^)^2^. Thus, we decided to use subscript “ini”, shorthand for initial states. The notation for the initial states of [Ca^2+^]_i_ and [Ca^2+^]_SR_ becomes [Ca^2+^]_i_^ini^ and [Ca^2+^]_SR_^ini^ throughout the paper.

2. In Figure 2, from context it seems that plots in panels A-D are calculated directly from the LRM, not simulations, but this is unclear. Please clarify in the text.

We added a sentence at Line 172 – 173 to clarify this.

3. The methods describe an Eqn S5 in the Supplement, but this equation is not included in the Supplement.

Sorry about this omission. We added a section at the end of the Supplement that includes Eqn S5.

4. This may be an issue with the journal manuscript submission site, but the quality of all of the figures is very poor and it is difficult to read the text in most of the figures.

It is possible that you didn’t click to download the full resolution figure files. There should be link for each figure on the figure page to download the original figure file. Our figures are all set to 300dpi. Some loss of quality seems to have occurred in the figures embedded in the manuscript file.

**Reviewer’s Comments to the Authors:**

**Reviewer #2**

Jin et al present a clever approach for simplifying a complex, non-linear, and stochastic mathematical model by using logistic regression for estimating the probability of spontaneous beats. This can provide computational advantages and is a good way of summarizing which variables are important in the observance of a phenomenon.
Thank you!
This is a good application of these sorts of simplified statistical models, and there’s a lot to like in the manuscript. Nonetheless, there are also weaknesses that should be addressed.
(1) I initially didn’t understand the simulation protocol, and I wasn’t sure how SR [Ca] could be considered a parameter, since this is a variable that changes with time in contemporary myocyte models. On the initial reading I was envisioning a cell that was being paced for roughly 100 beats, then the authors were observing whether spontaneous Ca release was observed after pacing was stopped. So the setup needs to clarify a that these cells are not regularly paced, and that they’ve created conditions where spontaneous release in the form of Ca waves always occurs (or at least I assume it does). Although these conditions are somewhat artificial for healthy myocytes, I can still see the value in the modeling.

Thank you for your suggestion. To clarify why we referred to [Ca^2+^]_SR_ as a parameter, it was originally stated in Results (Line 97) and Methods (Line 503), that [Ca^2+^]_SR_ and [Ca^2+^]_i_ refer as the initial state of [Ca^2+^]_SR_ and [Ca^2+^]_i_ state variables. We realized that the notation is confusing. The manuscript has been revised to use [Ca^2+^]_i_^ini^ and [Ca^2+^]_SR_^ini^ to refer to the initial states of [Ca^2+^]_SR_ and [Ca^2+^]_i_ state variables, respectively.

The cell is not paced in our simulation protocol. However, we did try to make the initial state of the cell close to the physiological diastolic state with two specific modifications. 1. the initial membrane potential is set to its resting value of -93.4 mV. 2. The initial NCX is partially allosterically activated (~20%-40%), as described in Chu et al. (PMID: 27377851). The only modification of the model to increase the propensity of ectopic beats is to increase RyR opening rate 4-fold. It has been described in Methods (Line 451 – 458).

We have not checked if all sampled MMI set always generated spontaneous Ca^2+^ release in the form of Ca^2+^ waves. When reading the simulation output of the cell model, we only focused on the membrane potential profile, checking if it crosses 0 mV (Line 111 - 112). It is likely that some MMI sets, having P(EB) = 0, do not generate Ca^2+^ waves at all, especially for the MMI sets having low [Ca^2+^]_i_^ini^ and low [Ca^2+^]_SR_^ini^. It would be possible to check if Ca^2+^ waves occur for each MMI set but this would not impact the results of the study. In fact, the advantage of our LRM framework for P(EB) is that although the EB-inducing complex spatial Ca^2+^ wave is mechanistically simulated in our myocyte model, we can forego the analysis of spatial Ca^2+^ signals and directly develop the LRM for P(EB) based on the membrane potential profile generated from the sample set of MMIs.

We did describe the details of the simulation protocol in the Methods. However, to further clarify the simulation protocol, we added a subsection (Line 98 - 105) in the first paragraph in Results to briefly reemphasize the protocol, where we first introduced these simulations.

(2) The text is rather dense and sometimes difficult to understand. Overall, results are described too much from a mathematical perspective, in terms of the performance of the logistic model, and less from a biological perspective, i.e. these are the biological implications of the results. The authors do that from time to time, but there are still many dense and jargon-filled sentences.
As an example, in lines 281-285: “The normal GK1_sf PDF primarily coincides with a region of the MC curve where P(EB) < ~0.3. In contrast, the HF GK1_sf PDF primarily coincides with a region of the MC curve where P(EB) > ~0.4.”
In general, the authors should revise to minimize sentences like this that are all about model and focus more on the ones that explain the significance of changes in parameters such as GK1.

To reduce heavily mathematical language in the Results, we made edits at Line 211-212, Line 218-219, 252-255 and 303-308. However, because the primary contribution of this paper is proposing a new efficient approach to estimating P(EB) and the development of arrhythmia uncertainty analysis, we believe that some mathematical descriptions are still necessary.

(3) A related comment concerns the concept of entropy of distribuions, which is mentioned a few times. The first mention of this is on line 201, in describing a specific result, without any introduction. Most readers will be left wondering: why is entropy important?

We added some introductory sentences about entropy describing why its calculation is meaningful (Line 212-216). Entropy is one proper way to quantify the degree of uncertainty in the P(EB) distribution. Larger entropy means larger uncertainty. The entropy is more appropriate than the variance to quantify the uncertainty when the distribution is multimodal. For example, entropy of P(EB) is largest when it takes on a uniform distribution, indicating it is equally likely to take on any value between 0 and 1 (which means we are completely uncertain of its value). However, the variance of the bimodal distribution shown in Fig 3C has a larger value than that of the uniform distribution, and therefore does not correctly capture the relative uncertainty in P(EAD). This again shows that entropy is more appropriate than variance for uncertainty characterization in this study.

Minor comments:
(1) A couple of references are duplicates, e.g. 3 and 12, and 20 and 29

We have screened through all citations and removed duplicated citations.
(2) In the Discussion, line 368, where the authors state that this is the first simplified model of arrhythmia dynamics, that’s not quite true. Morrotti & Grandi built a logistic model of EADs (PMID: 28116246) and Varshneya et al recently applied this approach to a study of arrhythmias caused by drugs for Covid-19 (PMID: 33205613). There is still considerable value in the present work because this is stochastic and spatial models, whereas those other studies were deterministic models of a different arrhythmia mechanism, but still.

Thank you for pointing this out! We edited this sentence (Line 430) in the summary to be more specific and say that this is the first simplified model for estimating the probability of cellular arrhythmias events from a stochastic spatial myocyte model.
