# Supplementary material for: Estimating ectopic beat probability with simplified statistical models that account for experimental uncertainty
Source: PLoS Comput Biol. 2021 Oct 19;17(10):e1009536. doi: 10.1371/journal.pcbi.1009536 (PMC8577785; doi:10.1371/journal.pcbi.1009536)
Supplement: Attachment — Submitted filename: response letter.docx [file pcbi.1009536.s007.docx]

Thank you for the opportunity to submit a 2^nd^ revision of the manuscript “Estimating Ectopic beat Probability with Simplified Statistical Models that Account for Experimental Uncertainty”. We appreciate the time and effort involved in providing feedback on our manuscript and are grateful for the insightful comments. We tracked all changes in the revised manuscript. A point-by-point response to reviewer’s comments and concerns are provided below. For easier reading, we use black for original reviewer comments, blue for our responses, and green for changes that we have made to the manuscript. Line numbers indicated here correspond to the line numbers appearing in the revised manuscript (tracked version) under the All Markup mode.

Reviewer’s Comments to the Authors:

Reviewer #1:

The authors have addressed all of my concerns.

Thank you very much.

Reviewer #2:

Although the authors have improved the manuscript during, a few aspects unfortunately remain unclear. I am not trying to be a difficult reviewer, but I am somewhat concerned that, in its present form, only modeling specialists will appreciate the work. The field contains many quantitative physiologists who value modeling but do not code models themselves (think David Eisner, Karin Sipido, Steve Houser, Bjorn Knollmann, etc). At present, I suspect that these potential readers would be confused about the physiological (or pathophysiological) conditions being simulated by the authors, and they would therefore not really appreciate the manuscript.

Thank you for sharing this concern. We address it in further detail below.
Part of my own confusion stems from the fact that, based on Figures 1A, 2E, and 2F, it appears that every simulation contains at least a small DAD. This would therefore imply that every simulation results in a cellular calcium wave. But with healthy myocytes in an experimental setting, waves are only seen infrequently, even after beta-adrenergic stimulation. Therefore, conditions have been created to favor the formation of waves, and it’s not really accurate to say “close to the diastolic physiological state,” as in the reviewer responses.

In Figures 1A, 2E and 2F, it is correct that each realization generates either a DAD or an ectopic beat. It was not our intention for this to imply that cellular Ca^2+^ waves always occur. In fact, while the examples shown in Figs. 1 and 2 demonstrate DADs or ectopic beats, additional realizations within the same selected region of interest (i.e. the range of parameters and initial conditions explored) do not generate any depolarizing activity (i.e. membrane voltage remains at the resting potential). This tends to occur when [Ca^2+^]_SR_^ini^ and [Ca^2+^]_i_^ini^ are small, G_k1_ is large, and k_RyR_^+^ is small within the biophysically meaningful region of interest, which is now described explicitly within the text of Results (Lines 118 - 119) and Methods (Lines 526 - 535), rather than appearing in a Supplement table.

To clarify this point, we included 5 samples of simulations in which no depolarizing events occur in Fig 1A and added a more complete description of the different types of responses generated by the model in the caption of Fig 1A and in the text of Results (Lines 120-121, 172 - 174).

We agree that the physiological condition that corresponds to the state of the model (within the region of interest) is not that of a resting diastolic state, and is in fact a state that favors the generation of DADs and ectopic beats with higher propensity than would be observed at rest. We revised the manuscript to state this point more explicitly. We added several sentences in Results (Lines 101 – 104) and Methods (Lines 468 - 469) to explicitly state that model simulations were performed under conditions that favor the generation of DADs and ectopic beats.
The “close to the diastolic physiological state” stated in the previous reviewer response was not clearly stated and should have referred only to the initial membrane potential and initial state of NCX (but not to the RyR opening rate parameter).

I don’t mean to belabor a minor point, but since experiments generally have to create conditions that will favor the production of calcium waves and DADs, I think it’s important for the manuscript to explicitly state this. This is alluded to a little bit with the description of the increase in the RyR opening rate, but in general it requires considerable work by the reader to figure this out, as he/she will need to read the first paragraph of the results, then look at a couple different sub-sections of the Methods, and then even have to download the supplementary table to fully understand. These aspects can still be improved.

We appreciate the need to be explicit on this point and have revised the text (as described above) to clarify that the state of the model is adjusted to mimic physiological conditions that favor the occurrence of DADs and ectopic beats.

Some related, more specific questions that came up while trying to understand these issues.
(1) The reader has to do a lot of work to understand how the parameters of the default cell differ from those previously used in the Walker et al study (Ref 5). The first paragraph of the results mentions the 4 fold increase in the RyR opening rate, but then lines 446-449 in the Methods lists other changes following the study from Chu et al. And then the question becomes: when multiple simulations are run with the default parameters, what percentage result in a calcium wave and DAD? As mentioned, it appears from Figures 1A, 2E, and 2F that every simulation results in a DAD.
I think this type of information is important for the reader to understand that conditions have been created that favor the occurrence of calcium waves.

We agree with the concern of the reviewer. To clarify how model parameters were adjusted from those used previously by Walker et al, we briefly mentioned all the difference between the Walker et al model and the myocyte model presented in this paper in the first paragraph of Results (Lines 104 - 110). A fully detailed description of the differences is provided in the “Modified Walker Ventricular Myocyte Model” section of Methods (Lines 446 – 464).

We create a default parameter set. [Ca^2+^]_i_^ini^ and [Ca^2+^]_SR_^ini^ are set to their diastolic values (based on steady state 0.5 Hz pacing under β-adrenergic stimulation). P(EB) for this default parameter set is 0.76 predicted from the LRM and 0.74 obtained by 100 realizations by the myocyte model. The realizations, which did not generate ectopic beats, all generate DADs, thus, generating Ca^2+^ waves.

We now report P(EB) values corresponding to the default parameter set in the Methods (Lines 536 - 544).
(2) Relatedly, Table S1 contains useful information about the ranges over which the parameters are varied. The reader shouldn’t have to go digging for this information, it seems like it would be really easy to include these 8 numbers in the Methods. Right now Lines 514-518 just say “obtained from experimental data,” which most readers will find unsatisfying.
But even after examining Table S1, we still don’t have all the information we need. Is the “default” cell mid-way between the minimum and the maximum?

We agree that the values in S1 Table should be incorporated into the main text in Methods. We removed the S1 Table and now report these values more prominently in Results (Lines 118 - 119) and Methods (Lines 526 - 535).

We added a detailed discussion of the range selection for [Ca^2+^]_i_^ini^ and [Ca^2+^]_SR_^ini^ in the main text (Lines 526 – 531). The “default” cell selection is described in Methods (Lines 536 - 544).

An additional modification was made to the reported range of the MMI: k_RyR_^+^_sf and the baseline value of k_RyR_^+^. We changed the range of k_RyR_^+^_sf from 2.5 – 7.5 to 0.5 - 1.5, and simultaneously changed the baseline value of k_RyR_^+^ from 1 to 5. The changes together have no net impact on the underlying model parameters as compared to the previous version (i.e. the product k_RyR_^+^_sf* k_RyR_^+^ is unchanged), but help to improve clarity.
